# Supplementary material for: A Global Screen for Assembly State Changes of the Mitotic Proteome by SEC-SWATH-MS
Source: Cell Syst. 2020 Feb 26;10(2):133–155.e6. doi: 10.1016/j.cels.2020.01.001 (PMC7042714; doi:10.1016/j.cels.2020.01.001)

Q15904 | VAS1_HUMAN | ATP6AP1 ATP6IP1 ATP6S1 VATPS1 XAP3
Monomer MW [kDa]: 52.026 Monomer expected elution fraction: 46

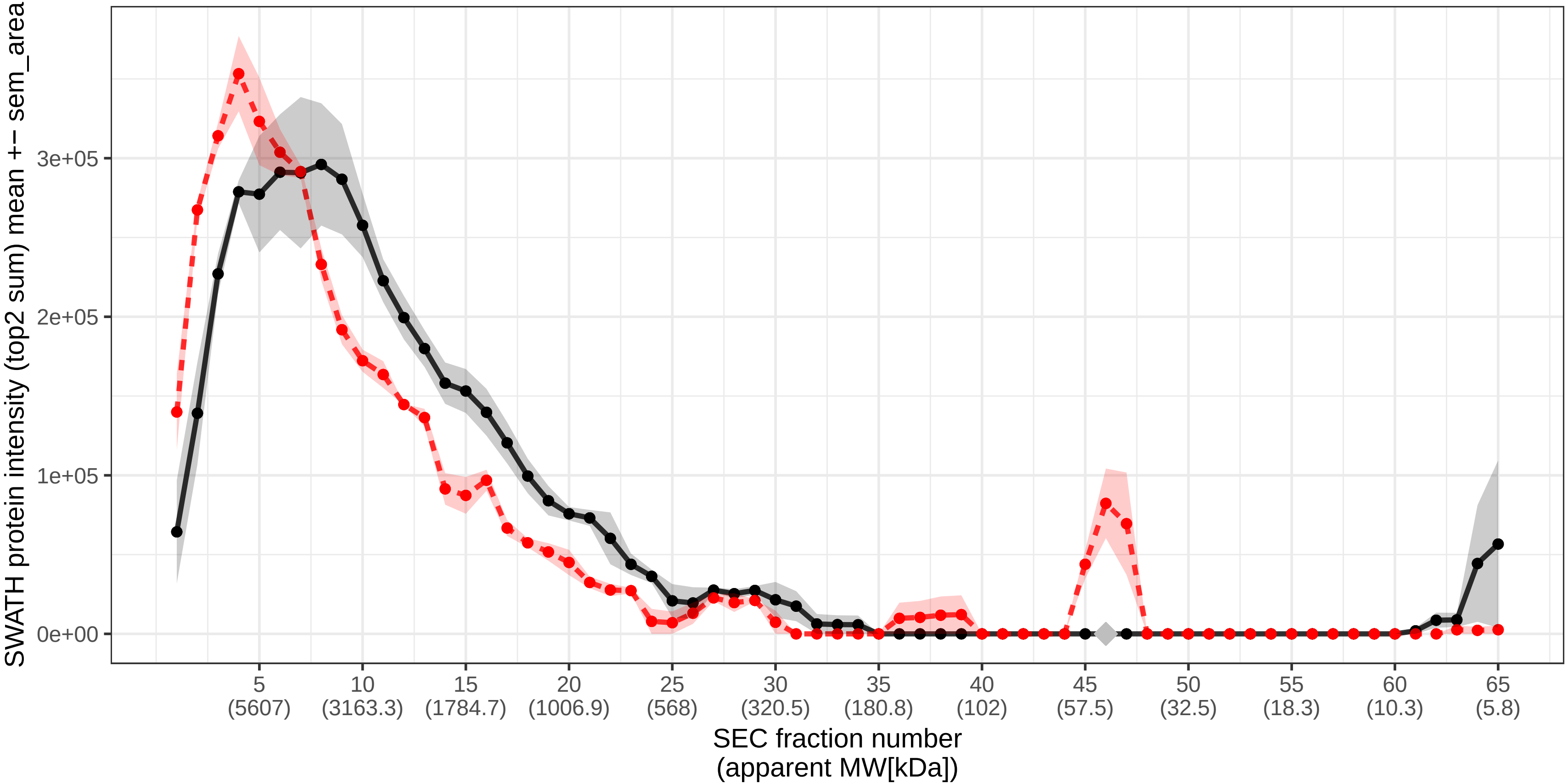

Supplement: Data S1. SEC-SWATH-MS Protein Chromatograms, Related to Figure 1 [file mmc6.zip › SECchrom_Q15904_VAS1_HUMAN_ATP6AP1_ATP6IP1_ATP6S1_VATPS1_XA.pdf]
